# The *Zea mays* mutants *opaque2* and *opaque16* disclose lysine change in waxy maize as revealed by RNA-Seq

**DOI:** 10.1038/s41598-019-48478-6

**Published:** 2019-08-22

**Authors:** Wei Wang, Suzhen Niu, Yi Dai, Mingchun Wang, Yan Li, Wenpeng Yang, Degang Zhao

**Affiliations:** 1 The State Key Laboratory Breeding Base of Green Pesticide and Agricultural Bioengineering, The Key Laboratory of Plant Resources Conservation and Germplasm Innovation in Mountainous Region (Ministry of Education), Guizhou University, Guiyang, 550025 China; 2 Guizhou Institute of Upland Food Crops, Guiyang Station for DUS Testing Center of New Plant Varieties (MOA), Guizhou Academy of Agricultural Sciences, Guiyang, 550006 China

**Keywords:** High-throughput screening, Gene expression analysis

## Abstract

In maize, *opaque2* (*o2*) and *opaque16* (*o16*) alleles can increase lysine content, while the *waxy* (*wx*) gene can enhance the amylopectin content of grains. In our study, *o2* and *o16* alleles were backcrossed into waxy maize line (*wxwx*). The *o2o2o16o16wxwx* lines had amylopectin contents similar to those of waxy line. Their nutritional value was better than waxy line, but the mechanism by which the *o2* and *o16* alleles increased the lysine content of waxy maize remained unclear. The *o2o2o16o16wxwx* lines and their parents on kernels (18th day after pollination) were subjected to RNA sequencing (RNA-Seq). The RNA-Seq analysis revealed 272 differentially expressed genes (DEGs). Functional analyses revealed that these DEGs were mainly related to biomass metabolism. Among them, in *o2o2o16o16wxwx* lines, 15 genes encoding α-zein were down-regulated, which resulted in the reduction of α-zein synthesis and increased lysine content; *lkr/sdh1* and *Zm00001d020984.1* genes involved in the lysine degradation pathway were down-regulated, thereby inhibited lysine degradation; *sh2*, *bt2* and *ae1* genes involved in starch metabolism were upregulated, leaded to wrinkling kernel and farinaceous endosperm. Our transcriptional-level identification of key genes responsible for increased grain lysine content and farinaceous endosperm formation following introgression of *o2* and *o16* alleles should promote molecular breeding for maize quality.

## Introduction

Maize (*Zea mays* L.), a very important food and feed crop, has a low protein nutritional value because it lacks lysine, a necessary amino acid in humans and monogastric animals. Maize line *opaque2* (*o2*) is a high-lysine mutant. The *O2* gene, which is located on the short arm of maize chromosome 7, encodes a leucine zipper family transcription factor containing a basic domain that activates the expression of 22 kDa α-zein and 15 kDa β-zein genes^1^ and can also directly or indirectly regulate other non-storage protein genes, such as *b-32* and *b-70*^2^. The *o2* mutant, which is widely used in genetics and breeding studies, has a grain lysine content of approximately 0.4%. Afterwards, several other mutations such as *floury1* ((*fl1,* (*opaque8*, *o8*; *opaque4*, *o4*))^3–5^, *opaque5* (*o5*)^6,7^, *proline1* (*pro1,* (*opaque6*, *o6*))^8–10^, *opaque7* (*o7*)^11–14^, *shrunken4* (*sh4*, (*opaque9*, *o9*))^15,16^, *opaque15* (*o15*)^17^, De*-B30^18^, Mc^18^, *floury2* (*fl2*)^19,20^ and *floury3* (*fl3*)^16,21,22^ were discovered. These mutants have been experimentally tried singly or in combinations, but resulted in severe yield losses due to negative effects of the individual mutation^23,24^. To enhance the germplasm resources of high lysine maize, we previously isolated a novel high lysine mutation from Robertson’s *Mutator* (*Mu*) stocks, which had opaque endosperm and was named *opaque16* (*o16*). The mutant line had a grain lysine content of over 0.36%. The corresponding gene was located on chromosome 8 L between molecular markers umc1141 and umc1121 within a 3 cM distance of umc1141^25^. The *o16* mutant is useful for germplasm improvement and quality breeding^26–28^, and its identification and study have contributed to high-lysine maize germplasm resources.

Despite the above improvements, high-lysine maize with a single gene mutation cannot meet the nutritional quality needs of food and food processing nor the lysine content requirements of livestock and poultry feed^29^. To further improve the lysine content of maize by marker-assisted selection (MAS), *o16* and *o2* alleles were pyramided. This approach yielded grains with a lysine content higher than 0.5%^25,30^, sufficient for the needs of human consumption. Konsam *et al*.^28^ then backcrossed *o16* alleles into *o2*-based parental inbreds (HKI161, HKI193-1, HKI193-2 and HKI163) of four quality protein maize (QPM) hybrids (HQPM-1, HQPM-4, HQPM-5 and HQPM-7) using marker-assisted backcross breeding (MABB). Compared with the recurrent parents, the contents of lysine and tryptophan increased by 76% and 91%, respectively. Average lysine and tryptophan contents of the hybrids increased by 49% and 60%, respectively, with maximum increases of 64% and 86%, respectively. Consequently, the pyramiding of high-lysine mutant genes can increase lysine and tryptophan contents to improve the nutritional quality of maize grains.

Waxy maize endosperm contains 95–100% amylopectin^31^. The *Waxy1* (*Wx*) gene is located on the short arm of chromosome 9, encodes granule-bound starch synthase I (GBSS I). The activity of GBSS I is significantly decreased in the *wx1* mutant, leading to the low level of amylose but high level of amylopectin in maize endosperm and pollen^32,33^. Waxy maize has excellent taste, texture and other culinary qualities, but its nutritional value is relatively low, and has a lysine content of only 0.24–0.34%^34^. To boost the lysine content of waxy maize, Zhang *et al*.^35^ used MAS to generate 18 inbred quality-protein maize lines containing *wx* and *o2* alleles. These lines had lysine contents of 0.36–0.54%, which was 1.15–27.06% higher than those of the original parents. Using MAS, Zhang *et al*.^36^ backcrossed the *o2* allele into waxy maize and increased lysine contents by 48.5–61.9%. Yang *et al*.^37^ incorporated the *o16* allele into waxy maize by crossing and backcrossing; they obtained four and three families, respectively, with lysine content increases of 18–28% compared with the original waxy maize lines. Also using MABB, Zhang *et al*.^38^ introgressed *o2* and *o16* alleles into *wxwx* lines and obtained three waxy maize lines containing *o2* and *o16* alleles, the mean grain lysine content was 0.62%, and the waxy property was equal to that of the recurrent parents, consequently meeting the food and feed requirements of humans, livestock and poultry. The pyramiding of high-lysine genes (*o2* and *o16*) and the waxy gene (*wx*) can therefore improve grain lysine content while retaining a constant waxy property^37–39^, thus yielding maize germplasm with good nutrition and flavour.

Single gene mutation and multiple-mutation pyramiding techniques can not only improve the nutritional quality of maize, but also affect body metabolism. Introgression of the recessive *o2* gene into common maize materials with different genetic backgrounds can cause different changes in transcription patterns^40^. The *o2* allele can also trigger physiological, biochemical, and proteomic changes in waxy maize^41^, *o2* introgression not only decreased the accumulation of various zein proteins, but also affected other endosperm proteins related to amino acid biosynthesis, starch-protein balance, stress response and signal transduction. In addition, the pyramiding of *o2* and *o7* alleles may affect amino acid metabolism, carbon metabolism, storage protein synthesis, transcription and translation, and signal transduction^42^. However, which physiological metabolic changes will be induced by the simultaneous introgression of *o2* and *o16* alleles into waxy maize is unknown, the molecular underlying mechanism of the *o2* and *o16* alleles introgression into waxy corn to increase the lysine content is still unknown.

To clarify the above questions, *o2* and *o16* alleles were backcrossed to waxy maize using MABB. Through target-gene foreground selection and genome background selection, mutant maize lines QCL8006_1 and QCL8006_2 containing three recessive *o2*, *o16* and *wx* gene mutations were obtained. To identify the molecular mechanism underlying the increased lysine content of waxy maize following introgression of *o2* and *o16* alleles, we carried out a transcriptome analysis of kernels on 18th day after pollination (18DAP) to examine transcriptional expression differences between *o2o2o16o16wxwx* mutants and their recurrent parent.

## Results

### Kernel characteristics and submicroscopic structure

**Figure 1:**
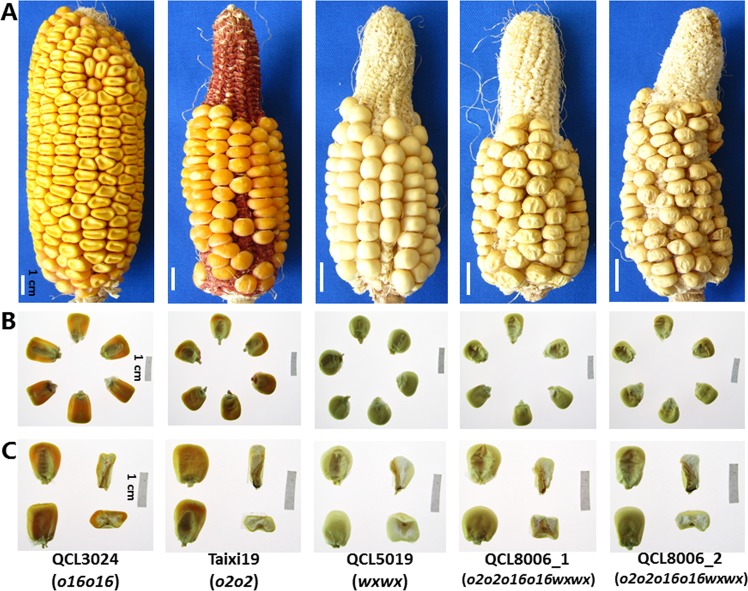
Phenotypic features of *o2o2o16o16wxwx* lines and their parents. (**A**) Photographs of intact ears taken under normal light. (**B**) Light transmission of mature kernels on a light box. (**C**) Cross-sections of mature kernels on a light box, Bars = 1 cm.

Grain phenotypes and endosperm cross-sections of *o2o2o16o16wxwx* mutant lines and their parent were observed under natural light, and the grain transparency was examined under projected light. Grain coats of *o2o2o16o16wxwx* mutant lines were non-glossy and displayed different degrees of wrinkling (Fig. 1A); the grains were completely opaque (Fig. 1B), with farinaceous endosperm and no full kernels (Fig. 1C). Grain coats of wild type (WT, CML535) were smooth and lustrous (Supplementary Fig. S1A), and the grains were vitreous (Supplementary Fig. S1B). Grains of *o16* mutant (QCL3024) were opaque with modifier, and grains of *o2* mutant (Taixi19) were opaque (Supplementary Fig. S1C). Grain coats of recurrent parent QCL5019 were smooth and lustrous, the grains were opaque and full with waxy endosperm.

**Figure 2:**
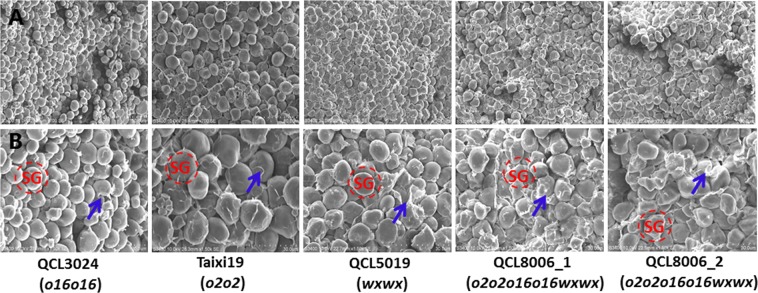
(**A**) Scanning electron micrograph for endosperms of QCL3024, Taixi19, QCL5019, QCL8006_1 and QCL8006_2 at 700× magnification, respectively. **(B)** Scanning electron micrograph for endosperms of QCL3024, Taixi19, QCL5019, QCL8006_1 and QCL8006_2 at 1500× magnification. SG, starch granules; Blue arrows, matrix protein.

Meanwhile, scanning electron microscopy revealed that the grain-endosperm starch granules of the *o2o2o16o16wxwx* mutants had an irregular shape and arrangement and an uneven volume and size, with a high density of matrix proteins that dispersed in the gap between starch granules. The grain-endosperm starch granules of recurrent parent QCL5019 were mostly ellipsoid or spherical; the matrix-protein density was low, and the starch granules were closely encapsulated (Fig. 2A,B).

### Changes of protein, starch and FAAs composition in *o2o2o16o16wxwx* endosperm

**Figure 3:**
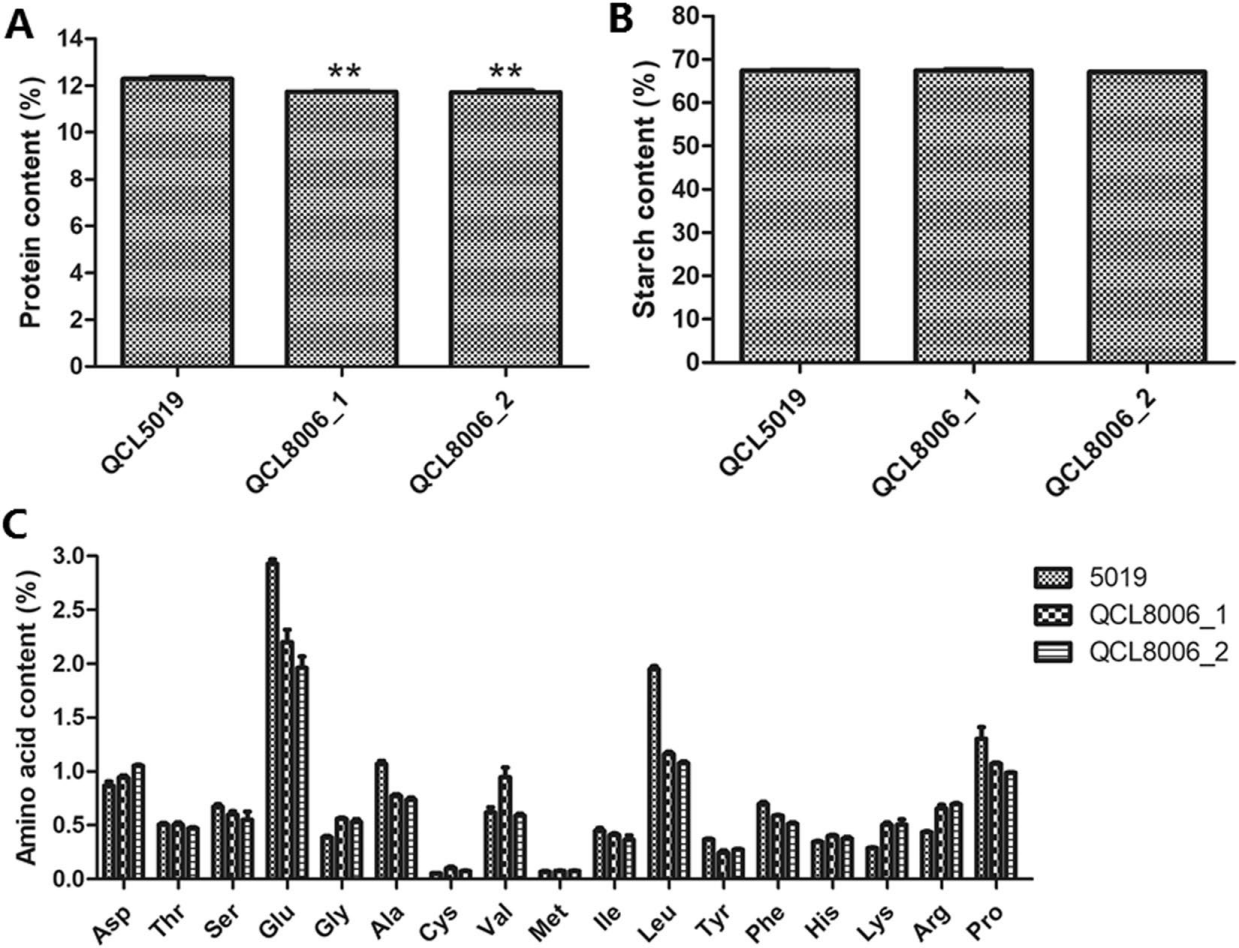
The contents of protein (**A**), starch (**B**) and 17 FAAs (**C**) in mature kernels of QCL5019, QCL8006_1 and QCL8006_2. *P < 0.05 and **P < 0.01.

To know biochemical differences between *o2o2o16o16wxwx* lines and their parent QCL5019, we measured protein, starch and free amino acids (FAAs) contents for their mature kernels. The total protein contents of QCL8006_1 and QCL8006_2 were 11.73% and 11.71%, respectively, which reduced by 4.52% and 4.65%, respectively, in comparison to QCL5019 (Fig. 3A). The total starch contents of QCL8006_1 and QCL8006_2 were 67.10% and 67.48%, respectively, and not significantly different from QCL5019 (Fig. 3B). The contents of 17 FAAs differed by varying degrees between recurrent parent QCL5019 and the *o2o2o16o16wxwx* mutants. Figure 3C shows that aspartate (Asp), glycine (Gly), cysteine (Cys), valine (Val), methionine (Met), histidine (His), lysine (Lys), and arginine (Arg) contents were higher in the mutants—significantly so in the case of Lys, Cys, Arg and Gly, which increased by 75.10%, 64.85%, 54.45%, and 40.33%, respectively. However, the contents of the remaining nine amino acids were lower in the mutants, and the total amino acid content also decreased slightly.

### The quality of RNA-seq and biological replicates

To dissect the regulator network of the *o2o2o16o16wxwx*, kernels (18DAP) of the *o2o2o16o16wxwx* and their parent lines were used for RNA-seq analysis. Each sample generated on average 23,648,912 clean reads (Supplementary Table S1), the average mapping ratio to reference gene and genome were 82.47% (Supplementary Table S2) and 91.12% (Supplementary Table S3). The average Q20 and Q30 (The percentage of the number of bases with Sequencing base mass value greater than 20 or 30 in the total number of bases in the original data) of all samples were 95.65% and 86.18%, respectively (Supplementary Table S4), which indicates that the sequencing data were of good quality.

According to our quantitative analysis (Supplementary Table S5), 29,137, 28,875 and 28,589 genes were respectively detected in QCL8006_1 and QCL8006_2 and their recurrent parent QCL5019; these numbers corresponded to more than 70% of the total number of genes (Supplementary Fig. S2), thus indicating that the sequencing saturation was in line with expected levels. Correlation coefficients among three sequencing repeats of QCL8006_1, QCL8006_2 and QCL5019 were 0.9516, 0.9948 and 0.9494, respectively (Supplementary Fig. S3); all of these values were higher than 0.92, which indicates that the transcriptome sequencing data of all three biological repeats in all samples were highly reliable and thus suitable for analysing differentially expressed genes.

### Identification of differentially expressed genes (DEGs)

**Figure 4:**
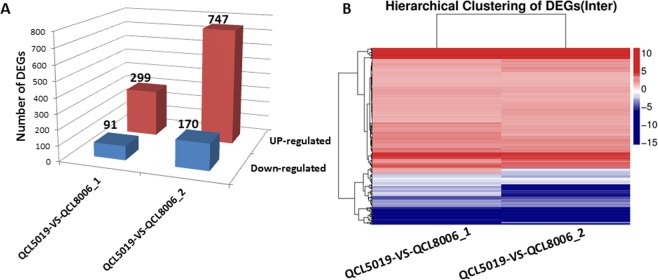
(**A**) The column diagram of DEGs for QCL5019 vs. QCL8006_1 and QCL5019 vs. QCL8006_2. X axis represents pairwise and Y axis means number of screened DEGs. Blue bar denotes down-regulated genes and red bar for the up-regulated. (**B**) The intersection heatmap of DEGs for QCL5019 vs. QCL8006_1 and QCL5019 vs. QCL8006_2. Gradient color barcode at the right top indicates log2 (FC) value (FC, Fold change of expression in triple recessive mutant vs waxy parent). Each row represents a DEG and each column represents one condition pairwise. DEGs with similar fold change value are clustered both at row and column level.

Gene expression differences between QCL8006_1 and QCL8006_2 and their recurrent parent QCL5019 were analysed using NOISeq software, and hierarchical clustering was also carried out. Compared with the recurrent parent QCL5019, 390 genes were differentially expressed in QCL8006_1, among which 299 were up-regulated and 91 were down-regulated. Meanwhile, in QCL8006_2, 917 genes were differentially expressed relative to QCL5019, and among them, 747 were up-regulated and 170 were down-regulated (Fig. 4A). In addition, to minimize differences due to different genetic recombination and genetic background, we examined the intersection of differentially expressed genes (DEGs) between QCL8006_1 and QCL8006_2 by COUNTIF function in Excel 2010 and the results show that there were 272 DEGs in common, 185 up-regulated, 79 down-regulated in both lines of QCL8006-1 and QCL8006-2, and other 8 up-regulated in QCL8006_1 but down-regulated in QCL8006_2 (Fig. 4B and Supplementary Table S6). It is thus evident that the expression trends of those 272 DEGs were almost identical, although there were obvious differences in DEGs detected between QCL8006_1 and QCL8006_2.

### GO annotation and KEGG analysis

**Figure 5:**
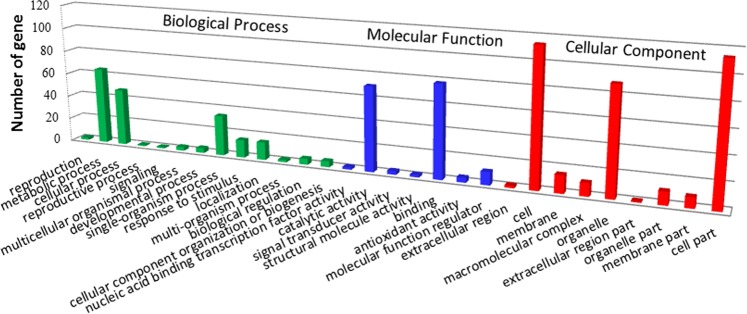
The GO analysis of differentially expressed genes. X axis represents GO terms. Y axis means number of DEGs (the number is presented by its square root value). All GO terms are grouped into three ontologies: brown is for biological process, orange is for cellular component and blue is for molecular function.

WEGO software^43^ was used to classify the differentially expressed genes into Gene Ontology (GO) functional categories related to biological process (BP), cellular component (CC), and molecular function (MF) (Supplementary Table S7). A total of 96 genes were classified into BP, mainly metabolic process (GO: 0008152), cellular process (GO: 0009987), single-organism process (GO: 0044699), localization (GO: 0051179), and response to stimulus (GO: 0050896). In terms of CC, 120 genes were classified mostly into categories related to cell (GO: 0005623), cell part (GO: 0044464), and organelle (GO: 0043226). A total of 117 genes were classified into MF categories, primarily those involving binding (GO: 0005488), catalytic activity (GO: 0003824) and molecular function regulation (GO: 0098772) (Fig. 5).

**Figure 6:**
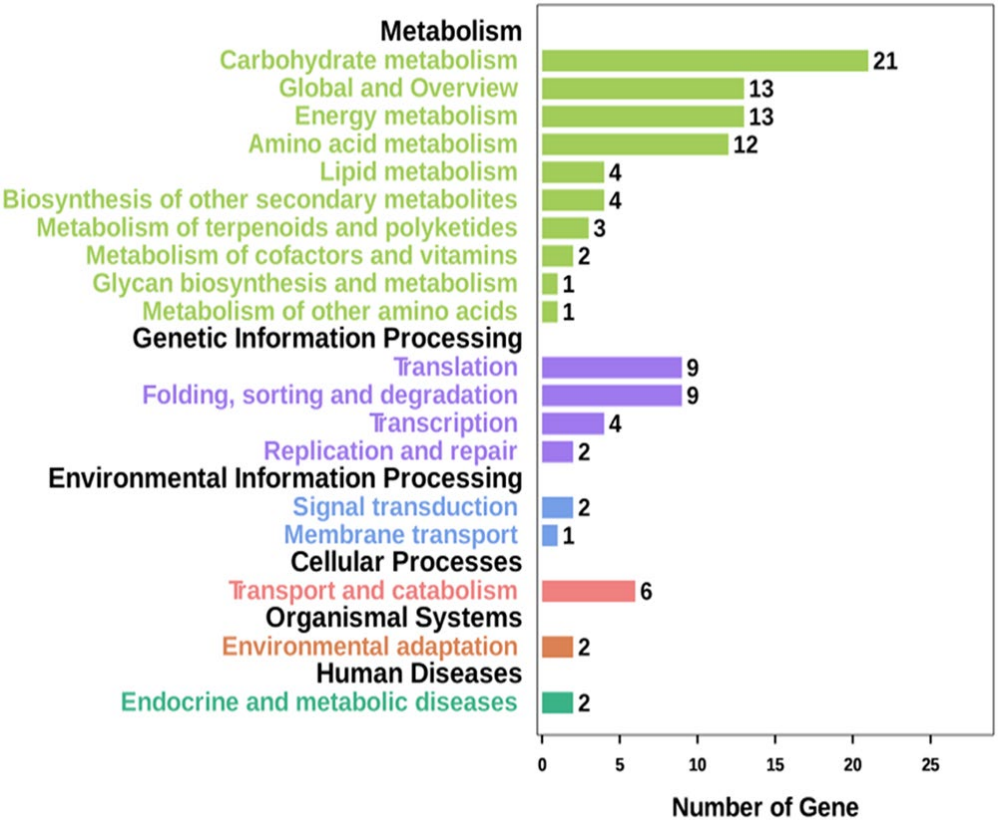
Pathway analysis of differentially expressed genes. X axis means number of DEGs. Y axis represents second KEGG pathway terms. All second pathway terms are grouped in top pathway terms indicated in different color.

To further functional characterization of DEGs, pathway analysis of DEGs based on the Kyoto Encyclopedia of Genes and Genomes (KEGG) database assigned 79 DEGs to 64 KEGG pathways (Supplementary Table S8 and Fig. 6). Seventy-five of these genes were annotated into metabolic pathways. Among genes related to metabolic pathways, 21 were related to carbohydrate metabolism, 13 to global and overview maps, 13 to energy metabolism, and 12 to amino acid metabolism. Of the 12 differentially expressed genes related to amino acid metabolism, seven were involved in amino acid synthesis and five participated in amino acid degradation.

### Amino acid metabolism

In regards to pathways related to amino acid synthesis, *Zm00001d014258.1* and *Zm00001d030557.1*, which encode glutamate-pyruvate aminotransferase and participate in the conversion of pyruvic acid to alanine in the alanine synthesis pathway, were up-regulated in *o2o2o16o16wxwx*. Other up-regulated genes were *Zm00001d016198.1* encoding glutamate-oxaloacetic transaminase, which converts oxaloacetic acid to aspartic acid in the aspartic acid synthesis pathway; *Zm00001d035443.1* encoding acetylornithine transferase in the conversion of *N*-acetyl-L-glutamate to *N*-acetyl-L-ornithine in the arginine synthesis pathway; and *Zm00001d027536.1* encoding serine acetyltransferase to transform L-serine to *O*-acetyl-L-serine in the cysteine synthesis pathway. Two genes were down-regulated: *Zm00001d025862.1* encoding threonine aldolase in the conversion of threonine into glycine and *Zm00001d027861.1* encoding serine-pyruvate aminotransferase in the conversion of glyoxalic acid to glycine (Table 1).

**Table 1:** Twelve DEGs involved in amino acid metabolism.

Gene ID	log2 Ratio	Description	Pathway
*Zm00001d047124.1*	−4.59	proline dehydrogenase EC1.5.5.2	proline metabolism
*Zm00001d027861.1*	−2.36	alanine-glyoxylate transaminase EC2.6.1.44	alanine biosynthesis
*Zm00001d025862.1*	−1.41	L-threonine aldolase EC4.1.2.5	alanine biosynthesis
*Zm00001d014258.1* *Zm00001d030557.1*	1.43 1.41	alanine transaminase EC2.6.1.2	alanine biosynthesis
*Zm00001d049380.1*	−1.13	4-aminobutyrate transaminase EC2.6.1.19	glutamate metabolism
*Zm00001d035443.1*	1.13	acetylornithine transaminase EC2.6.1.11	L-arginine biosynthesis
*Zm00001d016198.1*	1.41	glutamate-oxaloacetic transaminase EC2.6.1.1	L-aspartate biosynthesis
*Zm00001d027536.1*	1.98	serine *O*-acetyltransferase EC2.3.1.30	L-cysteine biosynthesis I
*Zm00001d052079.1*	−2.88	lysine-ketoglutarate reductase EC1.5.1.8	Lysine degradation
*Zm00001d020984.1*	−2.28	sarcosine oxidase EC1.5.3.7/EC1.5.3.1	Lysine degradation
*Zm00001d037498.1*	1.73	tryptophan aminotransferase related EC2.6.1.99	Tryptophan metabolism

In regards to amino acid degradation pathways, one gene was up-regulated: *Zm00001d037498.1* (*tar*) that encodes tryptophan aminotransferase and participates in the conversion of tryptophan to indole-3 pyruvate in tryptophan metabolism. The remaining four genes were down-regulated. *Zm00001d047124.1* encodes proline dehydrogenase for the conversion of proline to (*S*)-1-pyrroline-5-carboxylate in the proline degradation pathway. *Zm00001d049380.1* encodes 4-aminobutyrate transaminase and participates in the conversion of 4-aminobutnoate to succinate semialdehyde in the glutamate degradation pathway. *Zm00001d052079.1* encodes lysine-ketoglutarate reductase/saccharopine dehydrogenase (LKR/SDH1) (EC1.5.1.8) and thereby transforms DL-saccharopine to L-2-aminoglycolic acid-6-hemialdehydes in the lysine degradation metabolism pathway. Finally, *Zm00001d020984.1* encodes sarcosine oxidase (EC1.5.3.7/EC1.5.3.1) and participates in lysine degradation, which inhibits lysine degradation and thus increases lysine content.

### Carbohydrate metabolism

The 21 significantly differentially expressed genes involved in carbohydrate metabolism were mainly involved in starch/sucrose metabolism (ko00500), amino sugar/nucleotide sugar (ko00520), glycolysis/gluconeogenesis (ko00010), fructose/mannose metabolism (ko00051), and pentose/glucuronate interconversions (ko00040). Under starch and sucrose metabolism, genes *Zm00001d044129.1* (*shrunken2, sh2*) and *Zm00001d050032.1 (brittle2, bt2)* encoding ADP-glucose pyrophosphorylase (EC2.7.7.27) in the conversion of α-D-glucopyranose 1-phosphate to ADP-α-D-glucose in the starch synthesis pathway were up-regulated. Other up-regulated genes included *Zm00001d016684.1* encoding starch branching enzyme II (EC2.4.1.18) in the conversion of (1,4-α-D-glucosyl)_(n+1)_ to α(1,6)-α-D-glucosyl-(1,4)-α-glucan; *Zm00001d010801.1* encoding sucrose synthase (EC2.4.1.13) in the conversion of UDP-glucose to sucrose 6-phosphate in the sucrose synthesis pathway; *Zm00001d012433.1* encoding UDP-glucuronate decarboxylase (EC4.1.1.35) in the conversion of UDP-D-glucuronate to UDP-D-xylose in the xylose synthesis pathway; *Zm00001d015129.1* encoding galacturan 1,4-α-galacturonidase (EC3.2.1.67) in the conversion of pectate to D-galacturonate in the D-galacturonate synthesis pathway; and *Zm00001d021421.1* encoding UDP-glucose pyrophosphorylase (EC2.7.7.9/EC2.7.7.64) in the conversion of α-D-glucose-1P to UDP-glucose in the sucrose synthesis pathway. The gene *Zm00001d048099.1* encoding β-glucosidase (EC3.2.1.21) in the conversion of glucoside to α-D-glucose as well as the conversion of 1,4-β-D-glucan and cellobiose to β-D-glucose in the α-D-glucose synthesis pathway was down-regulated (Table 2).

**Table 2:** Twenty-one DEGs involved in carbohydrate metabolism.

Gene ID	log2 Ratio	Description	Pathway
*Zm00001d018966.1* *Zm00001d025753.1*	−3.23 1.55	chitinase EC3.2.1.14	Amino sugar and nucleotide sugar metabolism
*Zm00001d049380.1*	−1.13	4-aminobutyrate-pyruvate transaminase EC2.6.1.96	Butanoate metabolism
*Zm00001d031727.1*	1.56	L-iditol 2-dehydrogenase EC1.1.1.14	Fructose and mannose metabolism
*Zm00001d037278.1* *Zm00001d044754.1*	1.36 1.27	diphosphate-fructose-6-phosphate 1-phosphotransferase EC2.7.1.90	Fructose and mannose metabolism
*Zm00001d001999.1*	1.26	glucose-6-phosphate 1-epimrase EC5.1.3.15	Glycolysis/Gluconeogenesis
*Zm00001d024575.1* *Zm00001d051001.1*	−6.86 2.51	glyceraldehyde-3-phosphate dehydrogenase EC1.2.1.12	Glycolysis/Gluconeogenesis
*Zm00001d047893.1* *Zm00001d008318.1*	1.34 1.49	phosphoenolpyruvate arboxykinase EC4.1.1.49	Glycolysis/Gluconeogenesis
*Zm00001d045431.1*	1.67	phosphopyruvate hydratase EC4.2.1.11	Glycolysis/Gluconeogenesis
*Zm00001d046234.1*	−3.33	inositol oxygenase EC1.13.99.1	Inositol phosphate metabolism
*Zm00001d044129.1* (*sh2-shrunken2*)	1.42	adp glucose pyrophosphorylase, EC2.7.7.27	Starch and sucrose metabolism
*Zm00001d050032.1* (*bt2-brittle endosperm2*)	1.66	adp glucose pyrophosphorylase, EC2.7.7.27	Starch and sucrose metabolism
*Zm00001d048099.1*	−1.7	beta-glucosidase EC3.2.1.21	Starch and sucrose metabolism
*Zm00001d015129.1*	1.59	galacturan 1,4-alpha-galacturonidase EC3.2.1.67	Starch and sucrose metabolism
*Zm00001d016684.1* (*ae1-amylose extender1*)	1.16	starch branching enzyme II EC 2.4.1.18	Starch and sucrose metabolism
*Zm00001d010801.1*	8.55	sucrose synthase EC2.4.1.13	Starch and sucrose metabolism
*Zm00001d012433.1*	1.74	UDP-glucuronate decarboxylase EC4.1.1.35	Starch and sucrose metabolism
*Zm00001d021421.1* (*ugp2*)	1.31	ugp2-UDP-glucose pyrophosphorylase EC2.7.7.9, 2.7.7.64	Starch and sucrose metabolism

### qRT-PCR validation

**Figure 7:**
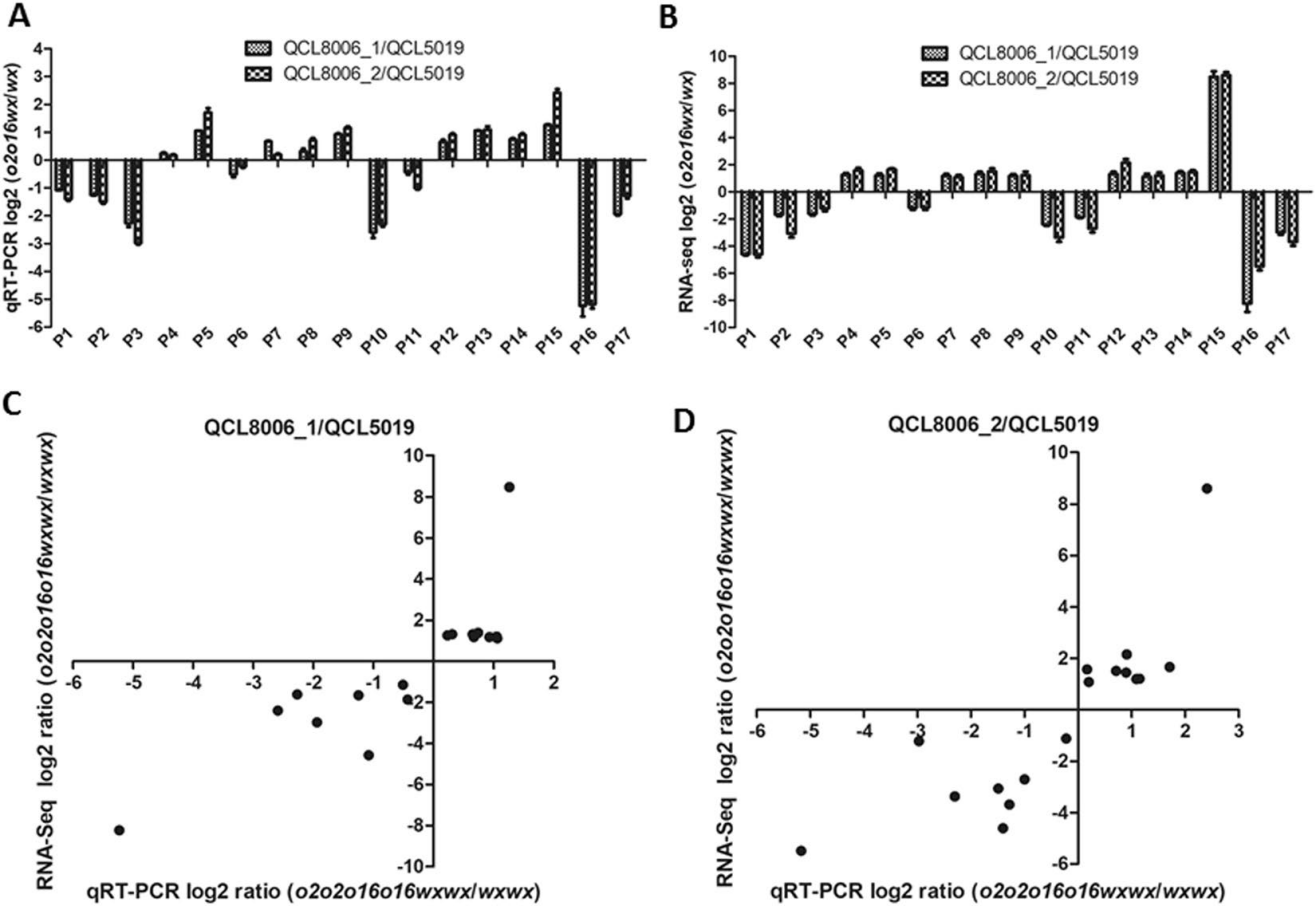
(**A**) The qRT-PCR log2 ratio of 17 DEGs for QCL5019 vs. QCL8006_1 and QCL5019 vs. QCL8006_2. (**B**) The RNA-Seq log2 ratio of 17 DGEs for QCL5019 vs. QCL8006_1 and QCL5019 vs. QCL8006_2. (**C**) The qRT-PCR validation of 17 DGEs for QCL5019 vs. QCL8006_1 identified by RNA-seq. Pearson’s r = 0.8409. (**D**) The qRT-PCR validation of 17 DGEs for QCL5019 vs. QCL8006_2 identified by RNA-seq. Pearson’s r = 0.8377. P1-P17, 17 DEGs.

The DEGs identified by RNA-seq were further validated by qRT-PCR. In this study, qRT-PCR (quantitative real-time polymerase chain reaction) verification was conducted for 17 DGEs, including 9 up-regulated and 8 down-regulated genes, involved in amino acid and carbohydrate metabolism (Supplementary Table S9 and Fig. 7A,B). The results showed that the expression patterns of these 17 genes were similar to those measured by transcriptome sequencing; the RNA-seq results showed high relevance to the qRT-PCR results (Person’s r = 0.8409, 0.8377) (Fig. 7C,D), indicating the reliability of the RNA-seq data.

## Discussion

In this study, we introgressed the *o2* and *o16* alleles into waxy maize line QCL5019 by using MABB technique, and two mutants (QCL8006_1 and QCL8006_2) (*o2o2o16o16wxwx*) were acquired. SNP (single nucleotide polymorphism) microarray analysis revealed that the recovery rate of the genetic background was over 95%; this level was higher than the theoretical genetic background recovery rate (87.50%), and nearly identical to that of the recurrent parent, indicated that the generated material was suitable for transcriptome analysis. On this basis, we analysed the transcription of the mutants (*o2o2o16o16wxwx*) and their recurrent parent at 18DAP by RNA-seq. We detected 272 DEGs in *o2o2o16o16wxwx* that were significantly differentially expressed relative to recurrent parent. These genes were mainly involved in cell, cell part, organelle, binding, catalytic activity, metabolic process, and cellular process. Among them, 75 genes were involved in metabolism pathways, including 21 participating in carbohydrate metabolism and 12 associated with amino acid metabolism.

Endosperm of *o2o2* and *o16o16* mutants is usually soft, fragile^1,2,25^ and that of waxy mutant was dark, smooth and waxy. In our study, the *o2o2o16o16wxwx* mutants, QCL8006_1 and QCL8006_2, were found to share similar ear phenotype with the recurrent parent QCL5019, but their kernel coats were non-glossy and wrinkled. In addition, their kernels were completely opaque, farinaceous and not full, suggesting that the *o2* and *o16* alleles may play a crucial role in the waxy genetic background^38^. SEM revealed that starch granules of the *o2o2o16o16wxwx* mutants had an irregular shape and arrangement and an uneven volume and size, with a high density of matrix proteins that dispersed in the gap between starch granules. Normally, maize starch granules were mostly ellipsoid or spherical, and tightly packed with protein bodies. However, in *o2o2o16o16wxwx* mutants, the shape and arrangement of starch granules were irregular and uneven, which may account for the wrinkling and fragile phenotype. Meanwhile, the protein matrix accumulates together rather than tightly surrounding the starch granules, may contribute to farinaceous endosperm.

In the present study, the total protein contents of QCL8006_1 and QCL8006_2 were reduced by over 4%, compared to QCL5019, which is consistent with previous studies in *o2o2o7o7* mutant^42^ and *o2o2wxwx* mutant^41^. However, the total starch content was not significantly changed between QCL8006_1, QCL8006_2 and QCL5019, which is inconsistent with the previous study^41^. In addition, to varying extents, the contents of free amino acids Glu, Leu, Ser, Ala, Ile, Tyr and Phe were lower in the *o2o2o16o16wxwx* lines than in the recurrent parent (*w*x*wx*), a result consistent with changes in amino acids due to introgression of the *o2* allele into *wxwx* maize observed by Zhou *et al*.^41^. In contrast, Lys, Cys, Arg and Gly contents increased significantly, especially Lys and Gly, this result was different from the observations of Zhou *et al*.^41^, who analysed *o2o2wxwx* mutants containing two recessive genes. The above differences may be related to the introgression of the high-lysine gene *o16* in our study and the different genetic backgrounds. RNA-seq analysis of DEGs uncovered 527 differentially expressed genes (448 up-regulated and 79 down-regulated) presented in QCL8006_2 but not in QCL8006_1. This disparity may be due to different genetic recombination and genetic background recovery rates. For example, the transcriptional profiles of *o2* and wild-type lines with different genetic backgrounds are different^40^. In the present study, we filtered out 272 differentially expressed genes shared between QCL8006_1 and QCL8006_2, which minimized the influence of such differences due to different genetic backgrounds and recombination and improved the efficiency of differentially expressed gene identification. Zhan *et al*.^44^ discovered that 1863 DEGs were detected in the endosperm of B73O2 versus B73o2 using RNA-seq of 15DAP endosperm, including 1024 DEGs that were downregulated and 839 DEGs upregulated in the *o2* mutant, and the result also showed the 494 DEGs that have been detected by at least one prior study^45–48^. Compared with those 494 DEGs, 66 DEGs were detected in the *o2o2o16o16wxwx* mutants versus *wxwx* parent, including 32 upregulated DEGs and 34 downregulated DEGs.

Lysine ketoglutarate reductase/saccharopine dehydrogenase (LKR/SDH) is a bifunctional lysine-degrading enzyme and a major regulator of free lysine content in plants. Kemper *et al*.^49^ found that the transcription level of LKR/SDH mRNA in maize *o2* mutants was decreased by more than 90% and that enzyme activity was significantly decreased, which reduced the degradation of lysine. Kawakatsu *et al*.^50^ reported that bifunctional rice OsLKR/SDH exists mainly in seeds and is directly regulated by the transcription regulatory factors RISBZ1 (rice seed *b*-Zipper 1) and RPBF (rice prolamin box binding factor) of seed storage protein genes; these authors also observed that decreases in RISBZ1 or RPBF lead to a decrease in OsLKR/SDH expression levels and an increase in the free lysine content of rice grains. In the present study, consistent with the above observations, *Zm00001d052079.1* encoding LKR/SDH and *Zm00001d020984.1* encoding sarcosine oxidase in the lysine degradation pathway were down-regulated, thereby inhibiting lysine degradation and increasing the grain lysine content of the *o2o2o16o16wxwx* lines.

Hartings *et al*.^42^ reported that transcription levels of 10 kDa γ-zein, 19 kDa and 22 kDa α-zein, and 27 kDa and 50 kDa γ-zein are significantly decreased in *o2o2o7o7* lines. Li *et al*.^51^ discovered that O2 not only directly binds to the promoters of known targets (22 kDa α-zein, 19 kDa α-zein and 14 kDa β-zein genes), but also to other zein genes, except for the 16 and 18 kDa zeins. Zhan *et al*.^44^ discovered that O2 directly regulates 23 zein genes, which were 13 genes of 19 kDa α-zein, 8 genes of 22 kDa α-zein, each one gene of 15 kDa β-zein, 18 kDa δ-zein, 27 kDa γ-zein and 50 kDa γ-zein. In our study, we obtained similar results, where 15 genes encoding α-zein were down-regulated in *o2o2o16o16wxwx* lines compared with *wxwx* parent (Supplementary Table S10), and of them 5 α-zein genes were detected by Li and Zhan *et al*.^44,51^. Therefore, the increased level of lysine in *o2o2o16o16wxwx* lines largely depends on the reduction of α-zein synthesis which excludes lysine, while several non-zein proteins which accounts for most of the higher percentage of lysine are associated with varying degrees of increased accumulation^49^. In the *o2o2o16o16wxwx* lines, *sh2* and *bt2* encoding adenosine diphosphate glucose pyrophosphorylase, and *ae1* encoding starch-branching enzyme were all upregulated, agreeing with data from Zhou *et al*.^41^. These changes are likely responsible for the formation of their wrinkling kernel and farinaceous endosperm, because of *sh2sh2* mutant with shrunken kennels, *bt2bt2* mutant with brittle kernels and *ae1ae1* mutant with wrinkled kernels.

Hidehiko *et al*.^52^ cloned the cDNA of alanine aminotransferase (AlaAT) from mature rice seeds and confirmed that AlaAT is involved in rice-seed nitrogen metabolism and storage protein synthesis. In addition, Shrawat *et al*.^53^ has shown that increased levels of alanine transferase can increase crop yields. In the present study, *alt4* and *Zm00001d030557.1* encoding alanine aminotransferase and *got3* encoding glutamic oxaloacetic transaminase were up-regulated in *o2o2o16o16wxwx* lines, which indicates that the introduction of *o2* and o*16* alleles into *wx* maize can increase the expression of alanine transferase (ALT) and glutamate oxalate transaminase (GOT) genes, promote amino acid transformation, facilitate dry matter accumulation and alleviate the contradiction between quality and yield.

**Figure 8:**
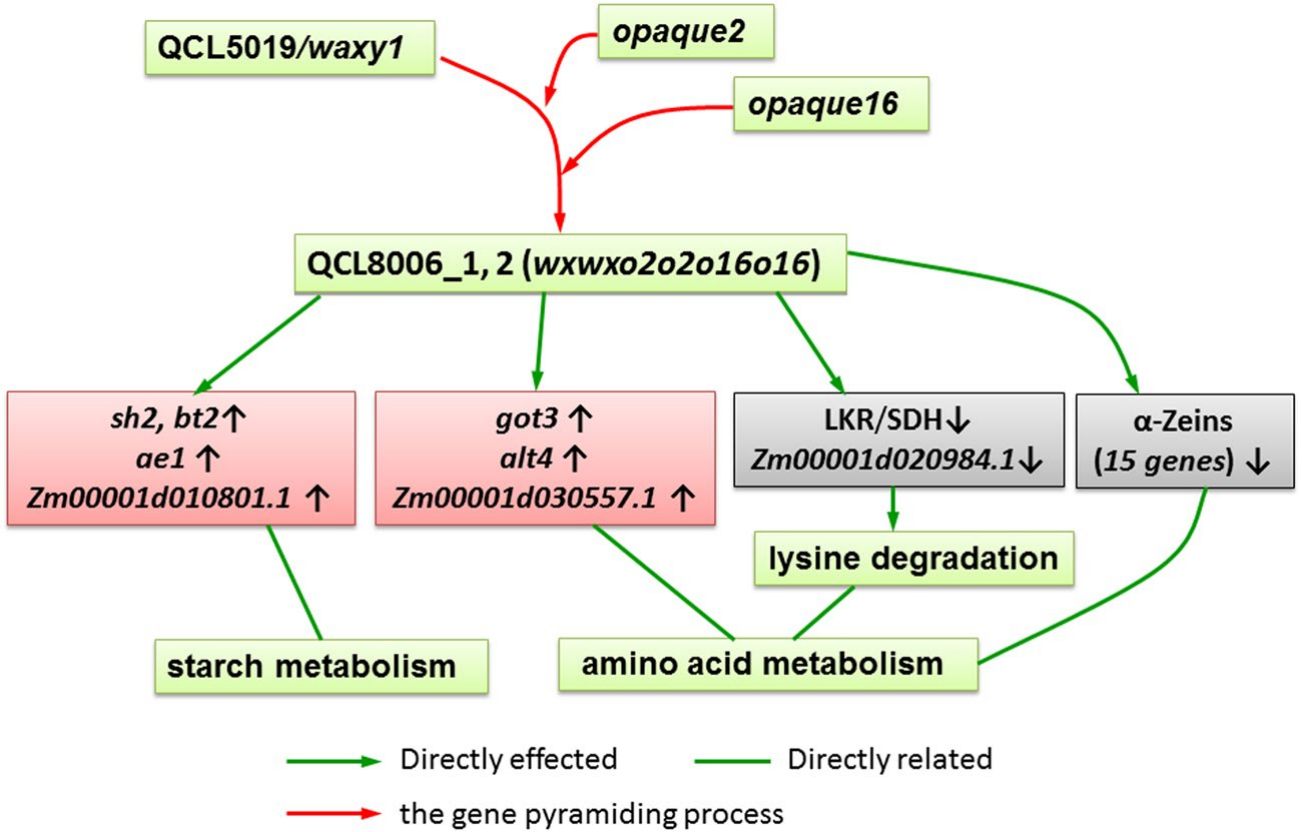
A proposed model of the regulatory network of waxy corn following the introgression of the *o2* and *o16* alleles. MAS, Marker-assisted selection; FS, Foreground selection; BS, Background selection; RNA-seq, RNA sequencing; ↑, -up-regualted; ↓, down-regulated.

In this study, the key genes responsible for the increase in lysine content and formation of opaque endosperm after introgression of *o2* and o*16* alleles into waxy maize were identified at the transcriptional level. Compared with the *wxwx* parent, in the *o2o2o16o16wxwx* lines, 15 genes encoding α-zein were down-regulated, which resulted in the reduction of α-zein synthesis and correspondingly in the increasement of non-zein, thereby increased lysine content. In addition, *lkr/sdh1* and *Zm00001d020984.1* genes involved in the lysine degradation pathway were down-regulated, thereby inhibited lysine degradation. Furthermore, *sh2*, *bt2* and *ae1* genes involved in starch metabolism were upregulated, leading to wrinkling kernel and farinaceous endosperm (Fig. 8).

## Conclusions

In this study, the *o2* and *o16* alleles were introgressed into the waxy corn using MABB, and QCL8006_1 and QCL8006_2 were acquired. Lysine contents of QCL8006_1 and QCL8006_2 were improved compared with that of recurrent parent. In the lines of QCL8006_1 and QCL8006_2, 272 genes were differentially expressed relative to recurrent parent by RNA-seq. GO and KEGG enrichment analyses revealed that these genes were mainly related to biomass metabolism. Among them, 12 genes were enriched for amino acid metabolic pathways, including down-regulated *lkr/sdh1* and *Zm00001d020984.1*, which inhibited the degradation of lysine. In addition, 15 genes encoding α-zein were down-regulated, which resulted in the reduction of α-zein synthesis, while several non-zein proteins which accounts for most of the higher percentage of lysine are associated with varying degrees of increased accumulation. Furthermore, 21 genes were related to carbohydrate metabolism; these included *sh2*, *bt2* and *ae1* genes upregulated, leaded to wrinkling kernel and farinaceous endosperm. These will help to uncover the transcriptional regulation mechanism in lysine content increased by *o2* and *o16* allele introgression into waxy maize.

## Materials and Methods

### Materials

A three-way F_1_ cross was performed using QCL3024 (*o16o16*) and Taixi19 (*o2o2*) as donor parents and QCL5019 (*wxwx*) as the recurrent parent. Using SSRs linked to *wx*, *o2* and *o16* as markers for *phi027*, *umc1066*, and *umc1121*, respectively, we selected individual plants with the *O2o2O16o16Wxwx* genotype from the F_1_ generation, and BC_1_F_1_ plants were obtained by backcrossing with QCL5019. In the BC_1_F_1_ generation, individuals with the genotype *O2o2O16o16wxwx* were selected, and BC_2_F_1_ plants were obtained by backcrossing with QCL5019. Next, BC_2_F_1_ generation individuals with the *O2o2O16o16wxwx* genotype were selected and then self-pollinated to obtain BC_2_F_2_ plants. In the BC_2_F_2_ generation, individual plants with the genotype *o2o2o16o16wxwx* were selected, and BC_2_F_3_ individuals were obtained by self-pollination. After analysing genetic background, lysine and waxy quality, and plant field performance, we selected two *o2o2o16o16wxwx* gene-pyramiding lines, which were named QCL8006_1 and QCL8006_2, following more than 20 generations of self-pollination to genetic stability (Supplementary Fig. S4). Lysine and amylopectin contents of grains were respectively 0.49% and 0.54% in QCL8006_1 and 99.87% and 99.25% in QCL8006_2 (Inspection and Testing Centre for Quality of Cereals and their Products, Ministry of Agriculture, Harbin, China). According to a 55 K SNP microarray analysis, recovery rates of the genomic genetic background of QCL8006_1 and QCL8006_2 were 95.22% and 95.12% respectively, higher than the theoretical background recovery rate (87.50%), because of selecting individual plants with high genetic background recovery rate using the whole-genome SSR markers in backcross generations, this result indicated that a comparative transcriptome analysis of *o2o2o16o16wxwx* mutant lines and the *wxwx* parent would be beneficial.

### Kernel characteristics and submicroscopic structure

Grains of the *o2o2o16o16wxwx* mutant lines and their recurrent parent were selected for analysis. The grains were checked for the presence of a smooth vs. wrinkled surface under natural light, and grain transparency was observed using a light box. After grains were cut open with a blade, the grain cross-sections were observed to determine the degree of starchiness or waxiness and then photographed.

Maize kernels were decapped and sliced in the centre of the kernel with a razor blade. A small piece of endosperm was isolated from each kernel, coated with platinum using an E-1010 ion sputterer, and observed by scanning electron microscopy. The submicroscopic structure was observed by the Guizhou Key Laboratory of Agricultural Biotechnology Guiyang, China (N26°30′14″ and E106°39′21″).

### Analyses of amino acid, protein and starch contents

Amino acid contents of mature maize grains were analysed using an automatic amino acid analyzer (InfratecTM1241 Grain Analyzer, Made in Sweden). Approximately 120–150 mg dried powder of each sample was weighed and transferred to a small tube, and 5 mL of 6 M HCl was added. Each tube was incubated at 110 °C in a water bath for 24 h; after cooling to room temperature, the pH was adjusted to 2.0 with approximately 4.8 mL of 6 M NaOH, and the solution was diluted to a volume to 100 mL using ddH_2_O. Each sample was then filtered through a 0.45 μm membrane into a 2 mL liquid chromatographic sample bottle and used for determination of the contents of 17 FAAs.

Protein and starch contents of maize mature grains were assayed by using near infrared analyzers. Three replicates of each sample were performed.

### RNA-seq library construction and sequencing

Eighteen days after pollination, total RNA of whole grains was isolated using a plant RNA kit (Omega) according to the “difficult sample” protocol of the manufacturer. The concentration and quality of each RNA sample was checked using a NanoDrop 1000 instrument. After rRNA removal and enrichment of mRNA using Oligo(dT) magnetic beads, mRNA was reverse transcribed into cDNA using random primer N6; this was followed by cDNA second-strand synthesis to form double-stranded DNA. Following adapter fusion, the fragments were amplified using specific primers by PCR. The PCR products were denatured into single strands, and a single-stranded DNA library was obtained by cyclization of the single-stranded DNA with a bridge primer. Transcriptome sequencing (single end 50 bp, SE50) was carried out on a BGISEQ-500 sequencing platform by Shenzhen Huada Gene Technology Co. Three replicates of each sample were sequenced.

### Identification of differentially expressed genes

Low-quality reads and reads containing adapters or having an unknown base percentage > 10% were removed from the original sequencing data. The resulting clean reads were aligned to the reference genome (B73 version4)^54^ using HISAT2^55^ with the following parameters: -p 8–phred64–sensitive -I 1 -X 1000, and aligned to reference gene using Bowtie2^56^ with the following parameters: -q–phred64–sensitive–dpad 0–gbar 99999999–mp 1,1–np 1–score-min L,0,-0.1 -p 16 -k 200, and to calculate the gene alignment rate. FPKM was calculated as the expression level of genes and transcripts by using RSEM software^57^. Differentially expressed genes were identified using NOISeq^58^ according to the following criteria: fold change ≥ 2 and corrected *P* ≤ 0.05.

### GO and pathway enrichment analysis of DEGs

Significant differentially expressed genes were mapped to the terms of the Gene Ontology database (http://www.geneontology.org/). The gene number of each GO term was calculated, and terms that were significantly enriched in differentially expressed genes were identified by the hypergenometric test using WEGO software^43^. The calculated *P* values were subjected to Bonferroni^59^ correction, and GO terms enriched in differentially expressed genes were identified based on a *P* ≤ 0.05. KEGG^60^ pathway enrichment was calculated in the same way as in the GO functional enrichment analysis. In this study, pathways, with a *P* value ≤ 0.05, were consider to be significantly enriched in differentially expressed genes.

### qRT-PCR validation

Seventeen DEGs were selected for qRT-PCR verification. The primers, shown in Table S7, were designed online (http://www.primer3plus.com/cgi-bin/dev/primer3plus.cgi), cDNA was synthesized using a reverse transcription kit (Thermo Scientific RevertAid First Strand cDNA Synthesis Kit, K1622) in a 20 μL reaction volume, and quantitative analysis was conducted on CFX Connect Real-Time PCR System (BIO-RAD, Hercules, CA) according to the method used by Liu *et al*.^61^. In a 10 μL reaction volume, using 2 μL of a twentyfold diluted cDNA solution, 5 μL of SYBR^®^ Select Master Mix (ABI), 0.5 μL of each primer (10 mM) and 2 μL ddH_2_O. The thermal cycling conditions were 2 min at 50 °C, 10 min at 95 °C, followed by 39 cycles of 20 s at 95 °C and 1 min at 60 °C. Three replicates were used for each sample and actin was used as an internal standard; the relative expression level was calculated using the 2^−ΔΔCT^ method.

### Accession codes

The raw data of RNA-seq reads were deposited in the National Center for Biotechnology Information (NCBI) database under accession number (PRJNA 512329), biosample accessions were as follows: SAMN 10666504, SAMN10666505, SAMN10666506, SAMN10666507, SAMN10666508, SAMN10666509, SAMN10666510, SAMN10666511 and SAMN10666512.

## Supplementary information

The *Zea mays* mutants *opaque2* and *opaque16* disclose lysine change in waxy maize as revealed by RNA-Seq

Table S7

Table S8

Table S5

Table S6
