# Supplementary material for: The Zea mays mutants opaque2 and opaque16 disclose lysine change in waxy maize as revealed by RNA-Seq
Source: Sci Rep. 2019 Aug 22;9:12265. doi: 10.1038/s41598-019-48478-6 (PMC6706447; doi:10.1038/s41598-019-48478-6)
Supplement: Supplementary file 1 — The Zea mays mutants opaque2 and opaque16 disclose lysine change in waxy maize as revealed by RNA-Seq [file 41598_2019_48478_MOESM1_ESM.pdf]

The *Zea mays* mutants *opaque2* and *opaque16* disclose lysine change in waxy maize as revealed by RNA-Seq

Wei Wang[1,2], Suzhen Niu[1], Yi Dai[1], Mingchun Wang[2], Yan Li[1], Wenpeng Yang[2,*] & Degang Zhao[1,2,*]

[1] The State Key Laboratory Breeding Base of Green Pesticide and Agricultural Bioengineering, The Key Laboratory of Plant Resources Conservation and Germplasm Innovation in Mountainous Region (Ministry of Education), Guizhou University, Guiyang 550025, China.

[2] Guizhou Institute of Upland Food Crops, Guiyang Station for DUS Testing Center of New Plant Varieties (MOA), Guizhou Academy of Agricultural Sciences, Guiyang 550006, China.

***** Correspondence: dgzhao@gzu.edu.cn (D.Z.), Tel.: +86-138-8501-2693; ywpmaize@126.com (W.Y.), Tel.: +86-135-1191-6286

Additional Information

Figure S1. Phenotypic features of *o2, o16* lines and wild type. **(A)** Photographs of mature kernels taken under normal light. **(B)** Light transmission of mature kernels on a light box. **(C)** Cross-sections of mature kernels on a light box, Bars = 1 cm.

Figure S2. The number of identified genes in each sample.

Figure S3. The heatmap of correlation coefficient values acrossing samples. a, b, c represents three biological replicates. Gradient color barcode at the right top indicates the minimum value in white and the maximum in blue. If one sample is highly similar with another one, the correlation value between them is very close to 1.

Figure S4. A breeding flowchart adapted for the introgression of *o2* and *o16* genes into the waxy line QCL5019. MAS, marker-assisted selection; FS, foreground selection; BS, background selection.

Table S1. Summary for RNA sequencing data of QCL5019, QCL8006_1 and QCL8006_2.

Table S2. Alignment statistic of reads aligned with reference gene.

Table S3. Alignment statistic of reads aligned with reference genome.

Table S4. QC20 and QC30 of RNA sequencing data for QCL5019, QCL8006_1 and QCL8006_2.

Table S5. FPKM of all gene for QCL5019, QCL8006_1 and QCL8006_2.

Table S6. 272 DEGs of QCL5019 vs. QCL8006_1 and QCL5019 vs. QCL8006_2.

Table S7. GO analysis of DEGs of QCL5019 vs. QCL8006_1 and QCL5019 vs. QCL8006_2.

Table S8. Pathway analysis of DEGs of QCL5019 vs. QCL8006_1 and QCL5019 vs. QCL8006_2.

Table S9. The qPCR primers of seventeen candidate DEGs for quantitative real-time PCR analysis.

Table S10. Fifteen DEGs involved in zein synthesis.

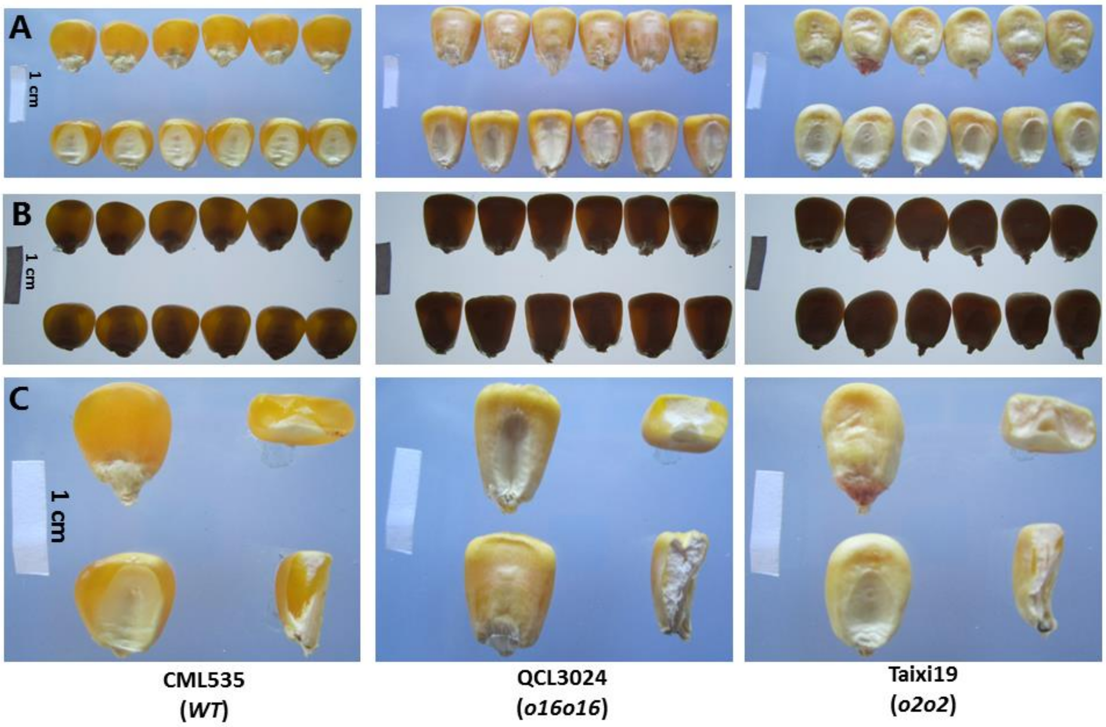

Figure S1 Phenotypic features of *o2, o16* lines and wild type. **(A)** Photographs of mature kernels taken under normal light. **(B)** Light transmission of mature kernels on a light box. **(C)** Cross-sections of mature kernels on a light box, Bars = 1 cm.

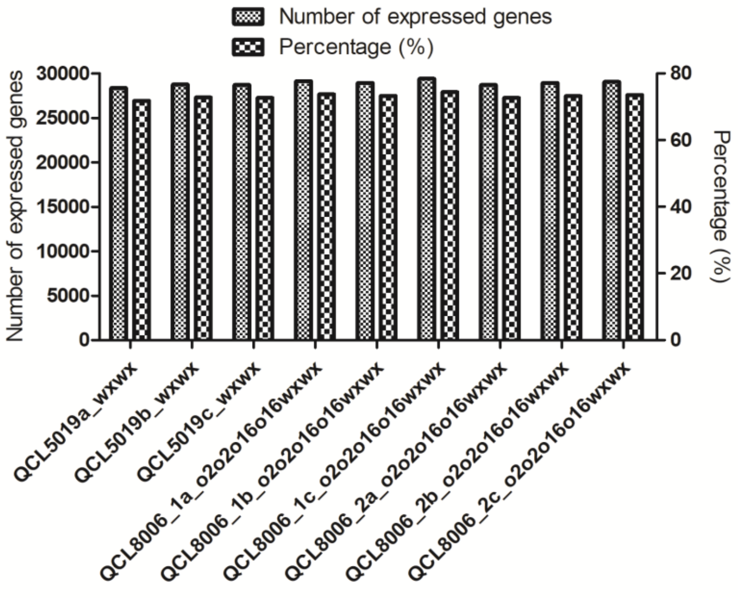

Figure S2. The number of identified genes in each sample.

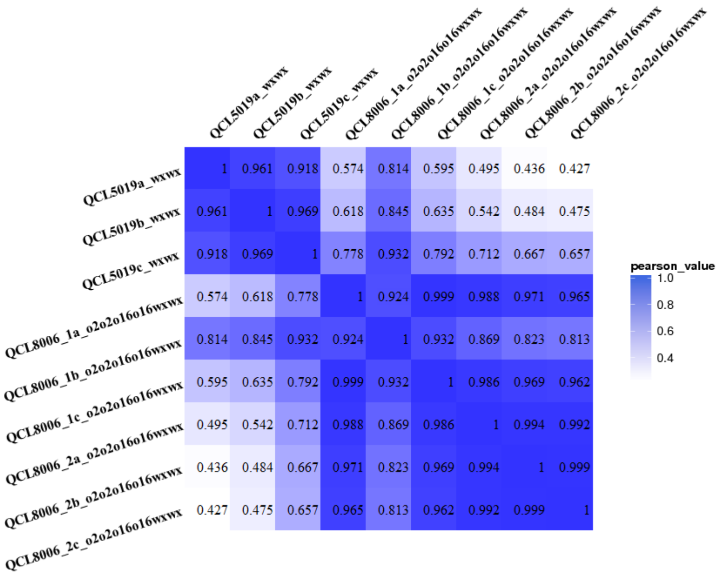

Figure S3. The heatmap of correlation coefficient values acrossing samples. a, b, c represents three biological replicates. Gradient color barcode at the right top indicates the minimum value in white and the maximum in blue. If one sample is highly similar with another one, the correlation value between them is very close to 1.

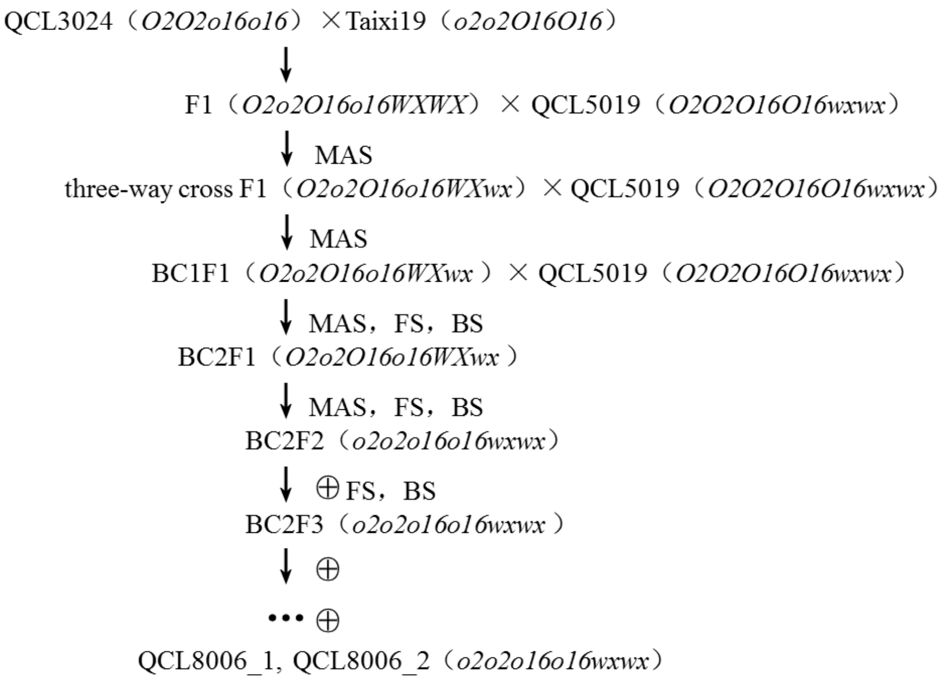

Figure S4. A breeding flowchart adapted for the introgression of *o2* and *o16* genes into the waxy line QCL5019. MAS, marker-assisted selection; FS, foreground selection; BS, background selection.

Table S1. Summary for RNA sequencing data of QCL5019, QCL8006_1 and QCL8006_2.

Sample	Sequencing Strategy	Raw Data Size (bp)	Discard Reads related to N	Discard Reads related to low qual	Discard Reads related to Adapter	Raw Reads Number	Clean Data Size (bp)	Clean Reads Number	Clean Data Rate (%)
QCL5019a_*wxwx*	SE50	1160101700	9908	53619	17823	23202034	1156034200	23120684	99.64
QCL5019b_*wxwx*	SE50	1202485350	41068	18814	44680	24049707	1197257250	23945145	99.56
QCL5019c_*wxwx*	SE50	1189537850	38545	8941	3984	23790757	1186964350	23739287	99.78
QCL8006_1a_*o2o2o16o16wxwx*	SE50	1196707250	41262	13306	90146	23934145	1189471550	23789431	99.39
QCL8006_1b_*o2o2o16o16wxwx*	SE50	1186843750	42694	27379	4358	23736875	1183122200	23662444	99.68
QCL8006_1c_o2o2o16o16wxwx	SE50	1205623100	41710	20380	50965	24112462	1199970350	23999407	99.53
QCL8006_2a_*o2o2o16o16wxwx*	SE50	1152633500	39116	13178	33018	23052670	1148367900	22967358	99.62
QCL8006_2b_*o2o2o16o16wxwx*	SE50	1206844550	5013	9821	6396	24136891	1205783050	24115661	99.91
QCL8006_2c_*o2o2o16o16wxwx*	SE50	1206836950	3780	4610	33964	24136739	1204719250	24094385	99.82

Note: Clean Data Rate (%)=Clean Reads Number/Raw Reads Number.

Table S2. Alignment statistic of reads aligned with reference gene.

Sample	Total Reads	Total Mapped Reads (%)	Unique Match(%)	Multi-position Match (%)	Total Unmapped Reads (%)
QCL5019a_*wxwx*	23120684	81.41	49.1	32.31	18.59
QCL5019b_*wxwx*	23945145	81.38	49.83	31.55	18.62
QCL5019c_*wxwx*	23739287	81.93	51.3	30.62	18.07
QCL8006_1a_*o2o2o16o16wxwx*	23789431	81.83	58.37	23.45	18.17
QCL8006_1b_*o2o2o16o16wxwx*	23662444	81.2	56.76	24.44	18.8
QCL8006_1c_*o2o2o16o16wxwx*	23999407	80.99	57.63	23.37	19.01
QCL8006_2a_*o2o2o16o16wxwx*	22967358	83.45	58.59	24.86	16.55
QCL8006_2b_*o2o2o16o16wxwx*	24115661	83.06	58.62	24.44	16.94
QCL8006_2c_*o2o2o16o16wxwx*	24094385	84.08	58.75	25.33	15.92

Note: Total Mapped Reads (%) = Unique Match (%) + Multi-position Match (%).

Table S3. Alignment statistic of reads aligned with reference genome.

Sample	Total Reads	Total Mapped Reads (%)	Unique Match(%)	Multi-position Match (%)	Total Unmapped Reads (%)
QCL5019a_*wxwx*	23120684	90.22	50.5	39.7	9.78
QCL5019b_*wxwx*	23945145	90.46	55.6	34.9	9.55
QCL5019c_*wxwx*	23739287	91.68	56.9	34.8	8.32
QCL8006_1a_*o2o2o16o16wxwx*	23789431	91.19	63.5	27.7	8.81
QCL8006_1b_*o2o2o16o16wxwx*	23662444	91.24	60.8	30.4	8.76
QCL8006_1c_*o2o2o16o16wxwx*	23999407	90.34	62.3	28	9.67
QCL8006_2a_*o2o2o16o16wxwx*	22967358	91.91	63.7	28.2	8.08
QCL8006_2b_*o2o2o16o16wxwx*	24115661	91.46	64.3	27.1	8.55
QCL8006_2c_*o2o2o16o16wxwx*	24094385	92.15	65.4	26.8	7.84

Note: Total Mapped Reads (%) = Unique Match (%) + Multi-position Match (%).

Table S4. QC20 and QC30 of RNA sequencing data for QCL5019, QCL8006_1 and QCL8006_2.

Sample	Clean Read1 Q20(%) >= 90	Clean Read1 Q30(%) >=	Clean Reads >= 20 (M)	Gene Unique Mapping Ratio(%) >= 80	Genome Mapping Ratio(%) >= 50
QCL5019a_wxwx	94.5 (Y)	84.69	23.12 (Y)	60.31 (N)	90.22 (Y)
QCL5019b_wxwx	96.5 (Y)	87.31	23.95 (Y)	61.23 (N)	90.46 (Y)
QCL5019c_wxwx	97.1 (Y)	88.61	23.74 (Y)	62.61 (N)	91.68 (Y)
QCL8006_1a_o2o2o16o16wxwx	96.8 (Y)	88.15	23.79 (Y)	71.33 (N)	91.19 (Y)
QCL8006_1b_o2o2o16o16wxwx	95.9 (Y)	86.12	23.66 (Y)	69.90 (N)	91.24 (Y)
QCL8006_1c_o2o2o16o16wxwx	96.3 (Y)	86.77	24.00 (Y)	71.16 (N)	90.34 (Y)
QCL8006_2a_o2o2o16o16wxwx	96.7 (Y)	87.71	22.97 (Y)	70.21 (N)	91.91 (Y)
QCL8006_2b_o2o2o16o16wxwx	97.1 (Y)	89.83	24.12 (Y)	70.58 (N)	91.46 (Y)
QCL8006_2c_o2o2o16o16wxwx	97.8 (Y)	91.16	24.09 (Y)	69.87 (N)	92.15 (Y)

Note: Q20, the percentage of the number of bases with Sequencing base mass value greater than 20 in the total number of bases in the original data; Q30, the percentage of the number of bases with Sequencing base mass value greater than 30 in the total number of bases in the original data.

Table S9. The qPCR primers of seventeen candidate DEGs for quantitative real-time PCR analysis.

NO.	Gene ID	Forward(5'- 3')	Reverse(5'- 3')	Amplicon length (bp)
P1	*Zm00001d047124.1*	AGCTAAACCCGACACCTTTC	AAGTGGAGGAGGATGCAATG	123
P2	*Zm00001d027861.1*	GATCCAAAGAGAGGCCAAAGA	GAAGGTCGAGGACAAACCTATT	122
P3	*Zm00001d025862.1*	CTCGGTGTGGTCTTATCTGTAATC	CAGGAACGAACACATCCAATAAAC	105
P4	*Zm00001d014258.1*	GTCACAGTCAGGGTATCAAAGG	GACTTGCTCCGTCTGTAATGAA	111
P5	*Zm00001d049380.1*	CGACAGAAATGGACGGGATAAA	CACTTCACTTCACGGGTTCTT	132
P6	*Zm00001d035443.1*	CGGAGTGGAACCAGACATAAT	CGTGGTCGCCGTAGTTTAT	113
P7	*Zm00001d016198.1*	TCGATGGAAGCTGATGGAATG	GTATATGGTAGCAGCAGGCTAAA	91
P8	*Zm00001d027536.1*	GCTGAGTGGTCTTTCACCAT	GCAGGATTACCTACAGCCATAC	104
P9	*Zm00001d052079.1*	CTGGTAGAGCTGGACTGATAGA	GAGATTGTCCCAGAGAGAGAAATG	96
P10	*Zm00001d020984.1*	AGTTCCACGGCACGAAAT	CACCTTCCAGTAGCAGATGAG	122
P11	*Zm00001d037498.1*	GCAGCCTCAGACATCTTTACT	GTAGCGAAGCCATGCAAATG	102
P12	*Zm00001d016684.1*	GATTTCATGGCCCTCGATAGAC	AGATAGCCCTCTCCTCCTAAAC	107
P13	*Zm00001d044129.1*	TTGTGAGGGTGATGGGATTG	CTCTACCACCAAAGCACCTATT	95
P14	*Zm00001d050032.1*	GAAAGAGGGTTCAGGCTTATCT	CTGGTATTGGCTTCTTGGTTATTC	105
P15	*Zm00001d010801.1*	CCAAGAAATTGCTGGAAGGTTT	GTACAGACCAGGCAGAGTAATG	99
P16	*Zm00001d024575.1*	CGACCGACAAGAACAGAAACTA	CTCTTTAGTCCACAACCACCTC	117
P17	*Zm00001d046234.1*	CTGTCGTTCCAAGTGTCCAT	GGGTACTCCTCTCAACTCTGTA	104

Table S10. Fifteen DEGs involved in zein synthesis.

gene id	log2Ratio	log2Ratio	description
Zm00001d048851.1 *(fl4 - floury4)*	-3.14	-3.91	encodes member of 19kD α-zein z1A-1 subfamily
Zm00001d030855.1 (az19D2 - alpha zein 19kDa D2)	-2.04	-9.34	alpha zein 19kDa D2 precursor
Zm00001d019155.1	-1.83	-5.79	zein-alpha A20-like precursor
Zm00001d019162.1	-1.583	-5.78	19 kDa zein A20
Zm00001d019160.1	-1.67	-6.06	zein-alpha A20-like precursor
Zm00001d019156.1	-2.17	-6.25	19 kDa zein A20
Zm00001d048847.1	-2.95	-8.03	zein-alpha Z4 precursor
Zm00001d048852.1	-2.19	-6.77	zein-alpha A30-like
Zm00001d049476.1	-2.77	-7.29	zein-alpha 19A2-like
Zm00001d048810.1	-8.50	-9.15	22 kD zein
Zm00001d048806.1	-7.53	-9.07	22kD alpha zein 5 precursor
Zm00001d048812.1	-9.93	-12.52	22kD alpha zein 4 precursor
Zm00001d048816.1	-7.54	-11.39	alpha-zein protein precursor
Zm00001d049243.1	-8.50	-11.31	22kD alpha zein 1 precursor
Zm00001d048813.1	-4.56	-12.46	zein-alpha 22C2 Precursor
